# Acoustic Estimation of the Direction of Arrival of an Unmanned Aerial Vehicle Based on Frequency Tracking in the Time-Frequency Plane

**DOI:** 10.3390/s22114021

**Published:** 2022-05-26

**Authors:** Nathan Itare, Jean-Hugh Thomas, Kosai Raoof, Torea Blanchard

**Affiliations:** Laboratoire d’Acoustique de l’Université du Mans (LAUM), UMR 6613, Institut d’Acoustique-Graduate School (IA-GS), CNRS, Le Mans Université, 72085 Le Mans, France; jean-hugh.thomas@univ-lemans.fr (J.-H.T.); kosai.raoof@univ-lemans.fr (K.R.); torea.blanchard@hotmail.fr (T.B.)

**Keywords:** drones, acoustic source localization, beamforming, microphone array, time-frequency representation

## Abstract

The development of unmanned aerial vehicles (UAVs) opens up a lot of opportunities but also brings some threats. Dealing with these threats is not easy and requires some good techniques. Knowing the location of the threat is essential to deal with an UAV that is displaying disturbing behavior. Many methods exist but can be very limited due to the size of UAVs or due to technological improvements over the years. However, the noise produced by the UAVs is still predominant, so it gives a good opening for the development of acoustic methods. The method presented here takes advantage of a microphone array with a processing based on time domain Delay and Sum Beamforming. In order to obtain a better signal to noise ratio, the UAV’s acoustic signature is taken into account in the processing by using a time-frequency representation of the beamformer’s output. Then, only the content related to this signature is considered to calculate the energy in one direction. This method enables to have a good robustness to noise and to localize an UAV with a poor spectral content or to separate two UAVs with different spectral contents. Simulation results and those of a real flight experiment are reported.

## 1. Introduction

In the last decade, unmanned aerial vehicles (UAVs) such as drones have been more and more frequently used for different purposes. It can be a very useful tool, for example for field or farming surveillance, package deliveries and film making. It could also potentially be a threat in some sensitive areas [1] or it could be used for illegal activities. Therefore, it is essential to be able to deal with these threats. The first step to deal with this issue is to know the exact location of the UAV. Several methods exist to localize an UAV, which can be efficient depending on the type of UAV and the conditions of flight [2,3]. Many studies have shown that localization methods using the noise of UAVs can be very effective [4,5,6]. Most methods use multiple microphone arrays and different algorithms to help estimate the direction of arrival (DOA) of the source. These algorithms can be separated in two categories: methods such as beamforming [7] and Capon [8], and subspace decompositions [9], such as Music [10], which sequentially scan the arrival directions to highlight the most energetic one and which differ mainly in their ability to separate spatially close sources and methods which will highlight the DOA by solving an inverse problem in a single pass from the raw recordings [11] delivered by the microphone array or from the computed time differences of arrival (TDOA) [12]. The latter methods can also exploit source sparsity [13,14,15]. All of these methods use the time signals recorded directly by the microphone array. In order to improve the signal-to-noise ratio (SNR), it is possible to take into account the acoustic signature of the UAVs. Indeed, many studies have shown that multi-rotor drones and Radio Controlled airplanes produce harmonic structured acoustic signals [16,17,18]. From this observation, the signal recorded by the microphone can be filtered by taking into account the fundamental frequency of the UAV signal. The fundamental frequency depends on the rotational speed of the rotors, and the complexity of the spectrum of the acoustic signal emitted will be influenced by the trajectory of the drone. Blanchard et al. [19] proposed an approach using time domain Delay and Sum Beamforming (DSB) with pre-filtered signal thanks to the detection of the fundamental frequency of a DJI phantom IV drone. For this purpose, a zero-phase digital filter is used to select the relevant frequency band by processing the signal in both forward and reverse directions. The disadvantage of this filter is that it is not easily implementable in real time, which is at least somewhat cumbersome in terms of computational resources. However, using DSB in the time domain seems relevant to us since it allows us to envisage a real-time implementation while presenting a good robustness to noise. Indeed, even if the technique requires focusing in a large number of directions, these operations can be parallelized if one has the means. It is true that using robust adaptive beamforming would also present an elegant solution for our purpose [20]. However, the technique requires inverting the interference-plus-noise covariance matrix, which may also include a regularisation step and the consideration of shorter snapshots to be averaged. The method is also imprecise when the positions of the antenna microphones are affected by errors, although a solution has recently been proposed [21]. These elements have, therefore, led us to keep the structure of the processing of [19], namely the implementation of DSB in the time domain, by completing it. The approach consists of using the time-frequency representation of the beamformer’s output while selecting only some harmonic contents related to the UAV’s signature and in calculating the energy related to this content. This process in the time-frequency domain avoids zero-phase filtering. A second objective of the paper is to go deeper into the fundamental frequency extraction process. In Ref. [19], this is carried out with an algorithm called Harmonic Product Spectrum [22] (HPS), which consists of multiplying compressed spectra at different compression rates. Several algorithms can be used to track the fundamental frequency of a modal structured signal such as Harmonic Line Association [23,24,25], or Spectral Harmonic Correlation (SHC) [26]. This tracking can be called pitch tracking and is also used for musical [27] or speech signals [26]. The paper is organized as follows: Section 2 describes the proposed method, the array configuration as well as the parameters that were used to carry out the simulations. Section 3 presents simulation results to establish the potential of the approach in the presence of one or two static sources and additive noise with comparisons to other methods. The directivity of the microphone antenna is also discussed. Section 4 is devoted to studying the performance of two tracking algorithms that are compared. The choice of the bandwidth surrounding the selected frequency is also discussed. Section 5 is dedicated to the consideration of motion. The localization performances are first analyzed for a simulated trajectory. Results from a drone recording are then presented. The influence of the area of the time-frequency plane used to compute the energy and deduce the localization of the UAV is also studied with the real trajectory. Section 6 finishes with a conclusion of the obtained results with perspectives.

## 2. Localization Method and Simulation Parameters

### 2.1. Delay and Sum Beamforming—Array Geometry

**Figure 1:**
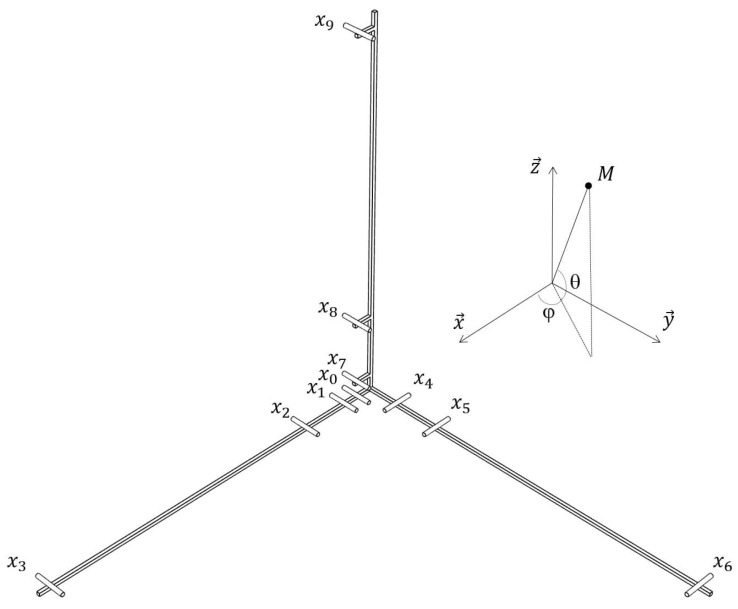
Coordinate system and microphone array used to localize the drone at M.

Delay and Sum Beamforming is a well-known method used with microphone arrays. This method makes use of the different information coming from the microphones. When a signal is sent by a source, each microphone receives this signal at a different time. By taking a reference microphone, it is possible to determine the delay between each microphone in the array and the reference microphone. The reference microphone is generally chosen at the center of the antenna. Time domain DSB is chosen for localizing the drone especially for its potential real-time implementation and its ability to operate robustly in the presence of noise. Figure 1 shows the used coordinate system and the microphone array designed by Blanchard et al. [19] to localize the drone. The geometry of the antenna was determined on the basis of several criteria: (1) to allow the localisation of a source in 3D (even if this is not the case of the present study for which the position in the azimuth-elevation plane is privileged), (2) to be sensitive to the range of frequencies emitted by the UAVs, specifically to be able to detect several harmonics of the blade passing frequency, (3) to comprise a limited number of sensors requiring few acquisition channels, and finally, (4) to be easy to transport and deploy. The respect of these constraints led to the development of a 3D antenna equipped with 10 microphones with 3 perpendicular axes, each comprising 4 microphones, one of which, the reference microphone $x_{0}$, is common to all three branches. The distances between each microphone and the reference are such that:

(1) $$\begin{array}{c} \left|\right|x_{1}\left|\right|=\left|\right|x_{4}\left|\right|=\left|\right|x_{7}\left|\right|=l_{1},\\ \left|\right|x_{2}\left|\right|=\left|\right|x_{5}\left|\right|=\left|\right|x_{8}\left|\right|=l_{2},\\ \left|\right|x_{3}\left|\right|=\left|\right|x_{6}\left|\right|=\left|\right|x_{9}\left|\right|=l_{3}, \end{array}$$

with $l_{1}=5$ cm, $l_{2}=20$ cm, and $l_{3}=110$ cm. The distance $l_{1}$ allows the maximum frequency of the bandwidth to be set according to the spatial Shannon criterion, while the distance $l_{3}$ contributes to setting the minimum frequency. This arrangement gives the antenna a bandwidth of [220.5, 3430] Hz, which is broad enough according to the DJI phantom IV signature. The geometry was, thus, chosen according to a frequency bandwidth-simplicity-portability compromise, which is obviously not the only one possible. Indeed, some studies lead to antennas with many more microphones (from 72 to 96 MEMS in [28]) with an arrangement obtained according to a hemispherical shape by optimising the gain in a given direction and the attenuation of the side lobes at a particular frequency using a genetic algorithm. A similar strategy is used in the development of a planar antenna to characterise the acoustic sources produced by a moving train [29]. It is also possible to optimise the geometry by constraining certain positions, for example by favouring intersections between the axes and the spiral path in a spiral antenna [30]. In contrast, some authors seek to optimise an antenna with only three sensors in the case of first-order steerable differential antennas by using the least squares method to match a directivity pattern in a given direction [31]. A recent study shows that a relevant geometry with a low number of sensors can be obtained by using the time difference of arrival in the fitness function to be optimised by particle swarm [32].

In our study, the location is represented with an angle Ω=(φ,θ), where φ and θ represent the azimuth and the elevation, respectively. The source is searched on a grid of [−180, 180]° in azimuth and [0, 90]° in elevation. The delays used to focus the beamformer on the virtual point are:

(2) $$\tau_{i}=\frac{\left|\;|\right|x_{0}M||-\left|\right|x_{i}M||\;|}{c},$$

where $\tau_{i}$ is the sound propagation delay between the ith microphone located at point $x_{i}$ and the reference microphone, $\left|\right|x_{i}M\left|\right|$ is the distance between microphone *i* and the source (*M*), and *c* is the speed of sound. The DOA is found by taking the direction which gives the highest energy.

### 2.2. Description of the Proposed Approach

**Figure 2:**
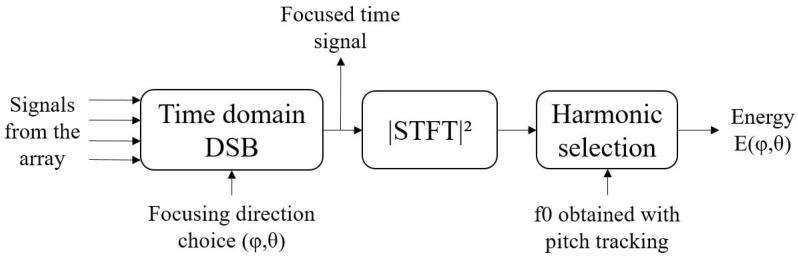
Synopsis of the proposed approach.

The process, applied to the signals acquired from a time section, is described in Figure 2. It is repeated for each chosen focusing direction (φ,θ) included in a grid whose resolution in azimuth and elevation is fixed a priori. The objective is to find the focusing direction that best corresponds to that of the drone from the information provided by the 10-microphone array. The direction that will prevail the decision is the one associated with the maximum energy E(φ,θ) of the focused time signal (using time domain DSB) for specific frequency bins. Indeed, the calculation of the energy is only based on certain components of the signal which are selected in the time-frequency plane (obtained by the Short Time Fourier Transform STFT of the focused signal). These are harmonics of a characteristic frequency of the UAV’s signature obtained by a pitch tracking algorithm run during each time slot of the reference microphone before focusing. Finally, all the time-frequency bins associated with these frequencies are taken to calculate the energy.

The approach enables to keep the robustness to noise of the DSB. It avoids pre-filtering each signal temporally before applying DSB, which requires a lot of processing [19]. Indeed, in order to keep the phase information intact, it is necessary to filter the signal a first time, time reverse the result then filter it again, and finally, time reverse the resulting signal [33].

### 2.3. Simulation of a Drone Signal

**Figure 3:**
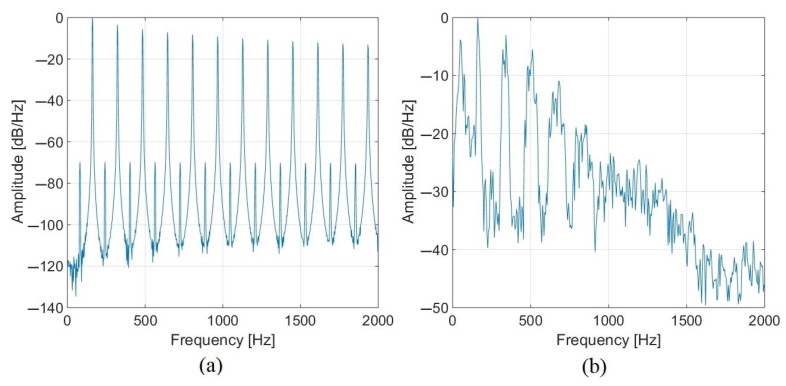
Power Spectral Densities of (**a**) the simulated drone signal provided by the model and (**b**) the real drone signal, both with $f_{0}=80.6$ Hz and $f_{bp}=161.2$ Hz.

The quadcopter DJI Phantom IV is used in the experiments. The aim is, therefore, to simulate the signals picked up by the microphone antenna, which should bear the signature of the quadcopter’s behavior. Two techniques are used for this purpose, one based on the signal model proposed in Refs. [19,34], inspired by Ref. [35], the other on numerically propagating a real signal to the virtual antenna. For both techniques, the acoustic source emitted by the UAV is assumed monopolar and the propagation is free-field. The real signal is obtained from the signals acquired by the array in an anechoic room: the UAV, held immobile by nylon wires while its motors are operated to move it, faces the antenna at a distance of 1.5 m. Thanks to beamforming, the measured signals are focused on the position of the UAV, providing the actual signal which can be propagated to any virtual array. Figure 3b highlights the Power Spectral Density (PSD) of such a simulated signal on the reference microphone. As for the model, it is built from the characteristics of the sound radiated by the UAVs described in the literature [16,17]. The signal emitted by the drone presents a harmonic structured spectrum with two types of harmonics highlighting two physical phenomena: the noise produced by rotation of the rotors (weak harmonics) and the noise produced by the blades (strong harmonics) giving the aerodynamic noise. The rotor frequency $f_{0}$ and the blade passing frequency $f_{bp}$ are related according to:

(3) $$f_{bp}=N_{b}*f_{0},$$

where $N_{b}$ represents the number of blades. The drone is equipped with two blades, so strong even harmonics with predominant amplitudes are present as well as weak odd harmonics with weaker amplitudes. Equation (4) gives the signal $p_{i}\left(t\right)$ received by the ith microphone using the model:

(4) $$p_{i}\left(t\right)=\sum_{n=1}^{N_{h}}\underset{\mathrm{weak}\;\mathrm{harmonics}}{\underset{︸}{\beta \frac{cos(2\pi \left[2n-1\right]f_{0}\left(t-|\right|x_{i}M||\left(t\right)/c))}{4\pi ||x_{i}M\left|\right|\left(t\right)}}}+\underset{\mathrm{strong}\;\mathrm{harmonics}}{\underset{︸}{\alpha_{f_{0}}\left(n\right)\frac{cos(2\pi \left[2n\right]f_{0}\left(t-|\right|x_{i}M||\left(t\right)/c))}{4\pi ||x_{i}M\left|\right|\left(t\right)}}}.$$

where $N_{h}$ is the number of harmonics in the signal, β sets the constant amplitude of the weak harmonics, and $\alpha_{f0}\left(n\right)$ is used to model a logarithmic attenuation of the strong harmonics extracted from the PSD of a real drone signal. The parameters for this study are $\beta =10^{-1.5}$ and $\alpha_{f0}\left(n\right)=10^{\frac{1}{20}\left(-11.6log_{10}\left(2nf_{0}\right)+65.4\right)}$. Figure 3a shows the PSD of the signal model with $f_{0}=80.6$ Hz and $f_{bp}=161.2$ Hz. A comparison of the two simulated signals shows that both types of harmonics are clearly visible, but there are fewer harmonics when using the real signal. It can be noted that the model does not take into account the presence of the four motors, as shown by the bandwidth around the harmonics, which is constant in the model and not for the actual signal.

## 3. Simulation Results in the Static Case

### 3.1. Directivity Pattern of the Designed Antenna

**Figure 4:**
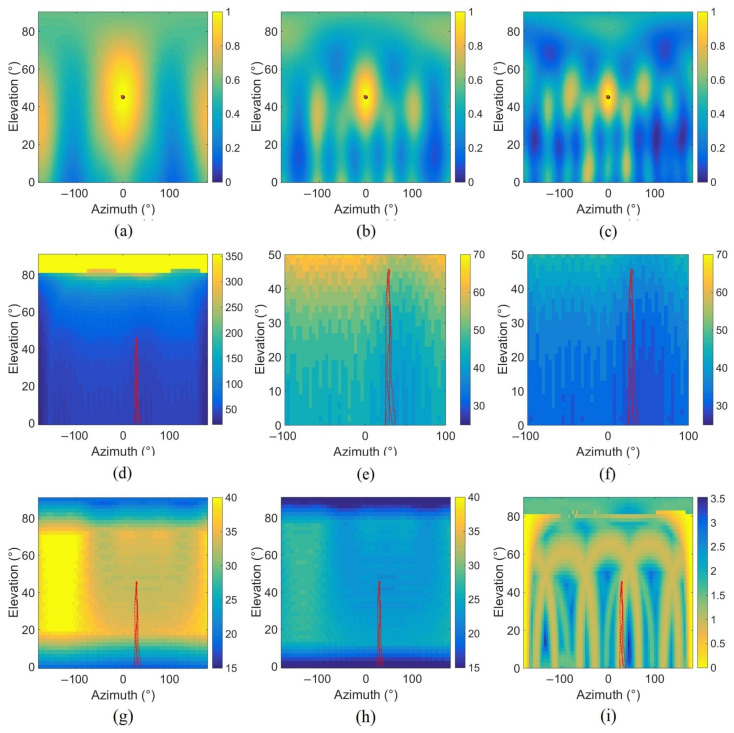
Directivity pattern of the array for (0°, 45°) direction and 175 Hz (**a**), 350 Hz (**b**), 525 Hz (**c**), with characteristics for all the directions in the azimuth/elevation plane: main lobe azimuth width (°) for 350 Hz (**d**), zoom for 350 Hz (**e**) and 525 Hz (**f**), main lobe elevation width (°) for 350 Hz (**g**) and 525 Hz (**h**), maximum side-lobe level (dB) for 350 Hz (**i**). The studied flight trajectories are in red.

The directivity of the antenna can have an influence on the localisation performance of the algorithms applied to the captured signals, especially in the presence of a disturbing source. This depends on the geometry of the antenna but also on the frequency studied and the focus point. To characterise the directivity pattern, the width of the main lobe in azimuth and elevation and the maximum level of the side lobes are used [36]. The lobe widths in azimuth and elevation are determined by the angular distance between the point of maximum energy and the point 3 dB below. The Maximum Side-lobe Level (MSL) is the level difference between the main lobe and the secondary lobe. The directivity pattern is obtained in the (φ,θ) plane from a monopole source simulated in one direction at 1 m [see Figure 4a–c for 175 Hz, 350 Hz, and 525 Hz]. The chosen frequencies correspond to the blade passing frequency measured in the presence of the UAV of Section 5 with two harmonics (4 ∗ $f_{0}$, 6 ∗ $f_{0}$). As the frequency increases, the main lobe is narrower and more secondary lobes appear for the same direction. The three parameters are calculated from the directivity pattern and plotted (see Figure 4d–i). The trajectories of the drone flight mentioned in Section 5.1 (solid line) and Section 5.2 (dotted line) are indicated on each map. Figure 4d–f show the main lobe azimuth widths calculated for the different source directions, for 350 Hz (d), with a zoom for the same frequency (e), and 525 Hz (f). It can be seen that for a source with an elevation higher than 80°, the azimuth width takes all the 360°. Everywhere else and especially around the trajectory, the width is in [38°, 60°] for 350 Hz (e) and in [29°, 39°] for 525 Hz (f). The width of the main lobe in elevation (g), (h) is different, it is in [35°, 40°] for 350 Hz (g) when the array focuses on [20°, 70°] in elevation and around 25° for 525 Hz (h). Figure 4i shows a MSL around 0 dB for azimuth around −180° and 180° for 350 Hz. Other directions provide a MSL between 1 and 3 dB with a periodic pattern. The trajectory considered gives an MSL around 1 dB, so it could be difficult to differentiate the secondary lobe from another source in the case of a multi-source flight. The cartography exhibiting the width of the main lobe in azimuth for frequency 525 Hz in the range [0°, 90°] is similar to Figure 4d as well as MSL to Figure 4i (350 Hz).

### 3.2. Simulations with One Source

**Figure 5:**
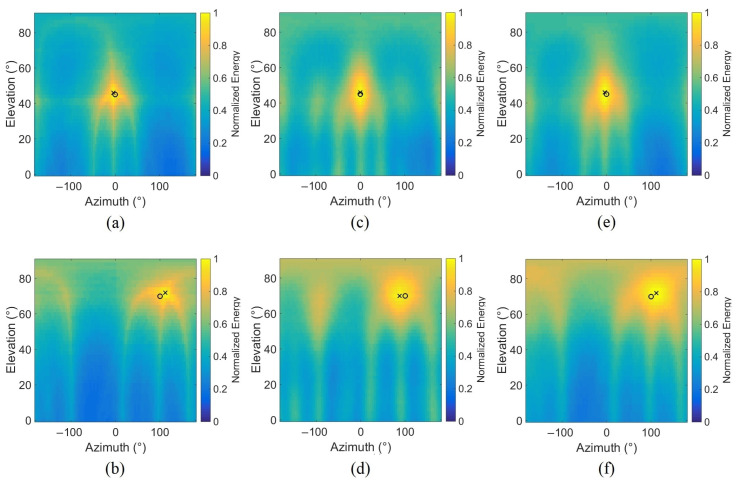
Normalized energy in the azimuth/elevation plane as a function of the position of the source [(0°, 45°) left, (100°, 70°) right], the method and the simulation technique used, (**a**,**b**): classical DSB, model; (**c**,**d**): classical DSB, real drone signal; (**e**,**f**): TFR, model (the circle represents the actual position of the virtual source, the cross represents the maximum energy position).

The proposed approach is first tested with a simple configuration involving a static source with a stationary rotor speed. This rotation speed is chosen to give a fundamental frequency of 80.6 Hz (which corresponds to a rotation speed of 4836 rpm). Some white Gaussian noise is added to the simulated signal with a SNR of 20 dB (standard power ratio between signal and noise). The performance of the approach to the noise will be evaluated later on. Two locations are tested, the first source is placed at (0°, 45°) which corresponds to 0° in azimuth and 45° in elevation and the second at (100°, 70°). The distance between the source and the antenna is 10 m. Beamforming is used to provide the energy in the azimuth/elevation plane with a grid resolution of (4°, 2°), respectively, in azimuth and in elevation. Energy is computed on a time signal of 2048 samples. Figure 5 shows the results of classical DSB using both source simulation techniques, based on the model [(a) and (b)] or the actual drone signal [(c) and (d)] for source positionning at (0°, 45°) [(a) and (c)] and (100°, 70°) [(b) and (d)]. As these results seem independent of the simulation technique, that of the approach using the Time-Frequency Representation (TFR) are only provided using the model at (0°, 45°) (e) and (100°, 70°) (f). For localizing a static drone, the signal model simulated according to Equation (4) thus seems to perform well. Five even harmonics are extracted from the TFR which means that the addition of the energy relative to the components $2*f_{0},4*f_{0},6*f_{0},8*f_{0},10*f_{0}$ is computed. All the bins contained in $\left[2*i*f_{0}-\frac{f0}{10};2*i*f_{0}+\frac{f0}{10}\right]$ intervals are selected with i=1,…,5, and the frequency bandwidth is $\Delta f=\frac{f_{bp}}{10}=\frac{2*f_{0}}{10}=16.12$ Hz. Indeed, when the SNR is low (≤40 dB), only the strong harmonics are present in the spectrum, so only even components are selected in this approach. The Fast Fourier Transform (FFT) is computed with 4096 points on Hanning windows of 2048 points with 512 points of overlapping. The maps represented in these figures are the calculated energies of the different angles Ω in the azimuth/elevation plane. Both locations show good accuracy for the classical DSB with a maximum energy (cross) close to the actual position of the source (circle). The TFR-based method shows similar results but with a little more energy where the source is located. This result shows that it is possible to locate a source by using only a restricted spectral content. It can be useful if some information is lost or if other sources overlap in another frequency range.

### 3.3. Simulation with Two Sources

**Figure 6:**
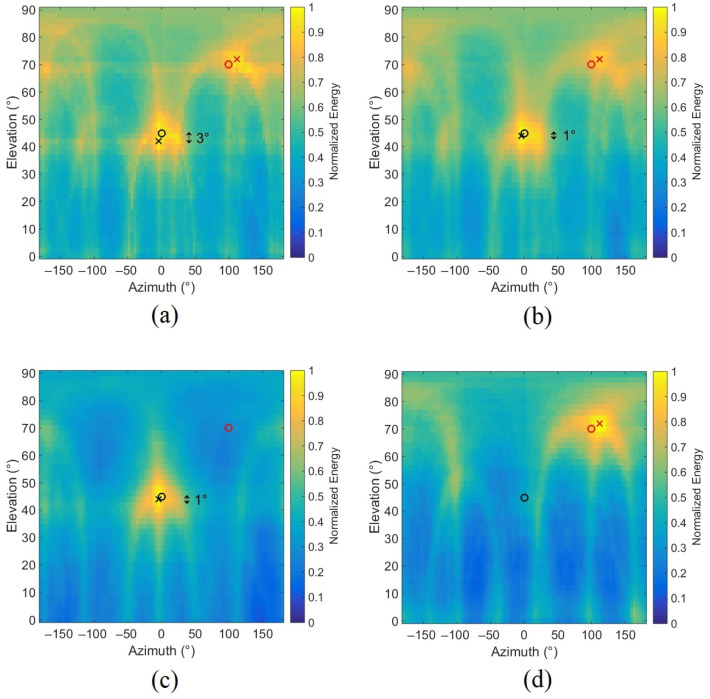
Normalized energy maps in the presence of two sources taking (**a**) the whole energy, (**b**) 5 harmonics of both sources in the TFR, (**c**) 5 harmonics of source 1 ($f_{01}=150$ Hz), (**d**) 5 harmonics of source 2 ($f_{02}=175$ Hz) (the circles and the crosses represent, respectively, the actual positions of the virtual sources and the positions of the two energy maxima).

Simulations conducted with two sources are reported in this subsection using the model signal defined in Equation (4). The same parameters are used in these simulations (resolution, SNR, $\left|\right|x_{0}M\left|\right|$) except for the fundamental frequency $f_{0}$, which is different here for each source. Generally, when two sources are present, they rarely simultaneously have the same spectral contents because of the different rotation speeds. Thus, two different fundamental frequencies are chosen close enough, for the first source $f_{01}=150$ Hz and for the second source $f_{02}=175$ Hz. The first source is located at (0°, 45°) and the second at (100°, 70°). Figure 6 presents the results for the classical DSB, for the TFR-based method taking frequency bins of the two sources in $\left[2*i*f_{01}-\frac{f_{01}}{10};2*i*f_{01}+\frac{f_{01}}{10}\right]$ and $\left[2*i*f_{02}-\frac{f_{02}}{10};2*i*f_{02}+\frac{f_{02}}{10}\right]$ with i=1,…,5, and for the TFR-based method using only one source. The results show that using the TFR increases the localization accuracy of the source placed at (0°, 45°) [3° in Figure 6a and 1° in Figure 6b]. Thus, depending on the location, the TFR-based method can improve location accuracy. The other interest of this approach is the separation of sources using their spectral contents. Indeed, when only the spectral content of source 1 is taken into account to calculate the energy, only source 1 is located in the energy map (Figure 6c). The same is true for the second source (Figure 6d); thus, it is a useful tool for separating multiple sources if each of them has a different spectral content.

### 3.4. Performance Evaluation

**Figure 7:**
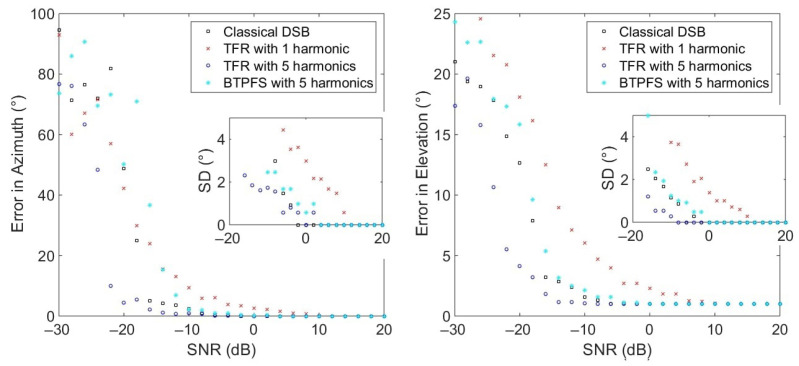
Evolution of the angle errors and standard deviations (SD) as a function of the SNR for the (0°, 45°) source with classical DSB, 1 harmonic considered in the TFR, 5 harmonics in the TFR and in the BTPFS.

The previous simulations have shown the performance of the TFR with different configurations and little noise (SNR = 20 dB). This subsection presents the evaluation of performance with different white Gaussian noise levels added to the simulated signals of the array. These signals are obtained by focusing signals measured in an anechoic room in the presence of the drone then propagating the beamformer’s output to the array at (0°, 45°) and 1 m. Figure 7 shows the angle errors with a varying SNR. In total, 50 virtual sources of 4096 samples with sampling frequency of 20 kHz were simulated for each noise condition and the shown result is the average of the direction estimations. Beamforming is processed with 2048 samples. The methods compared here are classical DSB, the TFR-based method with 1 and 5 harmonics, and Beamforming with Temporally Pre-Filtered Signals (BTPFS) using 5 harmonics [19]. Classical DSB gives an error of 0° in azimuth until −6 dB of SNR, whereas the approaches using the TFR with 1 and 5 harmonics start to rise at 10 dB and −14 dB, respectively. BTPFS with 5 harmonics start to have a rising error close to classical DSB with a faster rise. The standard deviation starts to increase in the same way as the mean errors. According to these results, it is better to use more harmonics in the TFR to have a better performance with lower SNR. The error in elevation is around 1° and starts to rise from −6 dB for classical DSB, from 10 dB for the TFR with 1 harmonic, from −14 dB for the TFR, and from −2 dB for BTPFS with 5 harmonics. The standard deviation is null and starts to rise when the error mean rises. For the azimuth and the elevation, the TFR-based method performs better with an error close to 0° until −16 dB in azimuth and 1° until −14 dB in elevation.

## 4. Pitch Tracking and Frequency Bandwidth

In the previous simulations, the fundamental frequency was assumed to be known, but this is not the case in reality. It is, thus, important to test and compare some algorithms to track the fundamental frequency. Two algorithms are presented here for pitch tracking: Harmonic Product Spectrum (HPS) and Spectral Harmonic Correlation (SHC). The way to choose the frequency bins around the frequency tracked for the energy calculation is also discussed.

### 4.1. Harmonic Product Spectrum

**Figure 8:**
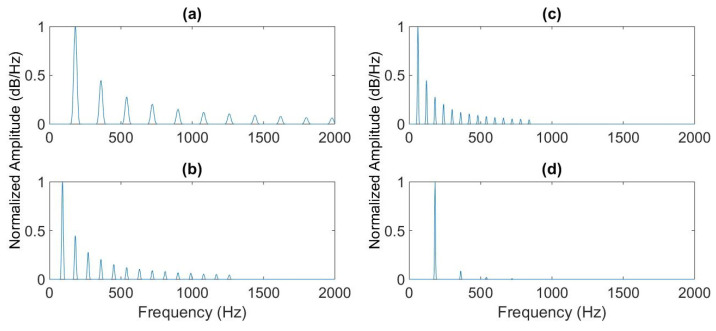
Part of the spectrum of the simulated drone signal (**a**), first (**b**) and second (**c**) compressions of this spectrum, and the product of the three spectra (**d**).

The HPS method developed by Schroeder et al. [22] exploits the harmonic structure of the signal spectrum. Since the harmonic signal has a spectrum with peaks multiple of the fundamental frequency, if the spectrum is compressed, the spectrum still has an harmonic structure but all the peaks’ frequencies are divided by two. When the spectrum is compressed multiple times, there is still a peak at the fundamental frequency. Therefore, the product of the different compressed spectra gives a high peak at the fundamental frequency and lower peaks at other frequencies. This process is performed according to Equation (5):

(5) $$f_{0}=\underset{f}{\arg\;\max}\sum_{k\in N^{*}}\log_{10}\left|X\left(kf\right)\right|,$$

where X(f) denotes the spectrum. Figure 8 shows the initial spectrum of the simulated drone signal with a fundamental frequency of 175 Hz, which corresponds to $f_{bp}$, and the two first compressed spectra. Because of the lower SNR, weak odd harmonics are not visible in the spectrum; therefore, the first frequency detected is $f_{bp}$ instead of $f_{0}$. The last subfigure shows the product of the spectra. The product gives a peak with high amplitude at the fundamental frequency and some peaks of lower amplitude elsewhere.

### 4.2. Spectral Harmonic Correlation

Spectral Harmonic Correlation (SHC) is based on a pitch tracking algorithm dedicated to speech signals called Yet Another Algorithm for Pitch Tracking (YAAPT) [26]. The idea of this algorithm is to calculate the correlation between one frequency and a chosen number of multiples of this frequency. When the correlation is strong, the result is close to 1 and the absence of correlation is linked to 0. Equation (6) describes how the SHC is obtained at time *t* and frequency *f* using the time-frequency representation S(t,f) and other parameters to calculate the correlation: $L_{w}$ is the window length used in the frequency domain. This means that for one frequency, the correlation is calculated between multiple windows of length $L_{w}$ that are equally spaced. Each windowed portion of the spectrum is selected with the coefficient *r* varying from 1 to $N_{H}+1$ the number of harmonics chosen for the correlation.

(6) $$SHC\left(t,f\right)=\sum_{f^{\prime }=-\frac{L_{w}}{2}}^{\frac{L_{w}}{2}}\prod_{r=1}^{N_{H}+1}S\left(t,rf+f^{\prime }\right).$$

**Figure 9:**
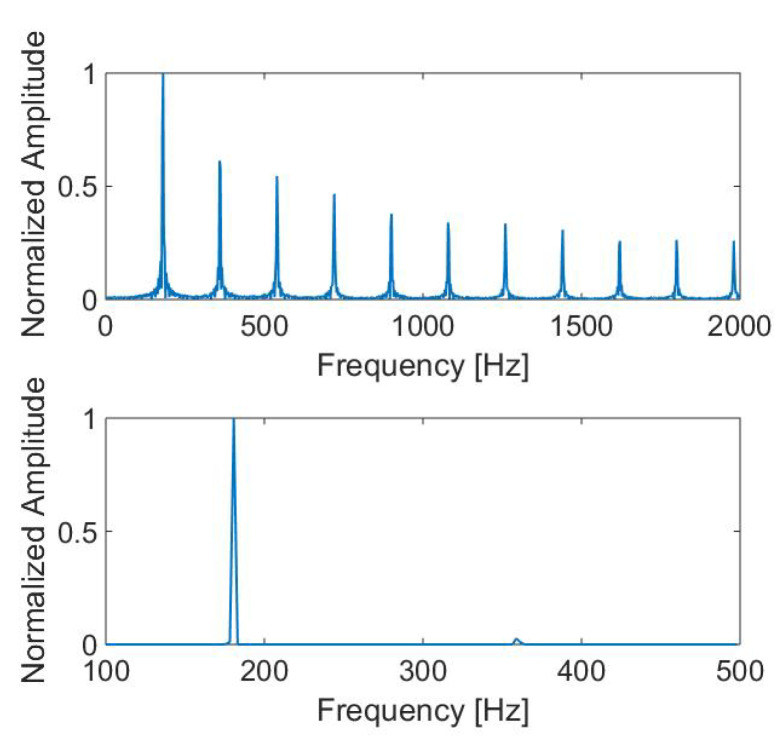
Magnitude of the Fourier transform of the simulated drone signal (**top**) and the SHC calculated with this spectrum (**bottom**).

Figure 9 shows the Fourier Transform’s magnitude of a simulated drone windowed signal centered at time *t* and the associated SHC calculation. For this simulation, $L_{w}=10$ Hz, $N_{H}=4$, and the calculation of the SHC is done between 100 Hz and 500 Hz because the blade passing frequency is in this interval for the DJI Phantom IV [34]. The SHC performs well and gives a correlation at this frequency ($f_{bp}=175$ Hz) and nowhere else.

### 4.3. Performance Evaluation

**Figure 10:**
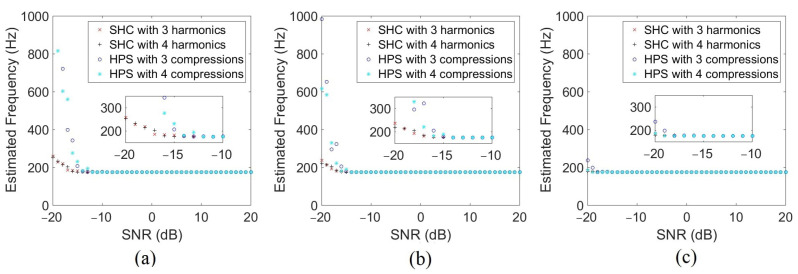
Evolution of the estimated blade passing frequency as a function of the SNR for a simulated source with HPS with three or four compressions and SHC with $N_{H}$ = 3 or $N_{H}$ = 4, with 2048 points (**a**), 4096 points (**b**), and 8192 points (**c**) for the FFT.

Just as the localization methods were tested with varying noise levels, the pitch tracking methods are evaluated here with different SNRs. Both methods use the spectrum for pitch tracking, so the performance depends on the number of points in the FFT, and on the parameters inherent to each method. Figure 10 presents the results for three different numbers of points for the FFT and with a varying number of harmonics in the SHC and compressions in the HPS. For each noise condition, 100 virtual sources have been simulated and the result shown is the average. The fundamental frequency of the simulated signal is $f_{0}=87.5$ Hz ($f_{bp}$ = 175 Hz). The lowest number of points 2048 enables to have an accurate blade passing frequency estimation until −14 dB of SNR for the HPS and −16 dB for the SHC. The SHC appears to perform better than the HPS in the presence of strong noise. It can also be seen that when the number of points is doubled, estimation performance allows for more unfavorable SNR levels of 2 dB. These results combined with results shown in Figure 7 give the simulation limits of the SNR for the localization methods presented in this paper.

### 4.4. Frequency Bandwidth around the Tracked Frequency

In the process of harmonic selection in the TFR, another influential parameter is the frequency of the considered bandwidth. Indeed, after the STFT, only some time-frequency bins are chosen to calculate the energy. The selected bins are at least those for which frequencies are equal to $f_{detect}*i$, $i=1,\ldots ,n_{h}$, with $f_{detect}$ the frequency detected by the pitch tracking algorithm and $n_{h}$ the number of harmonics chosen. $f_{detect}$ normally corresponds to $f_{bp}$. More frequency bins can be chosen in the neighborhood of $f_{bp}$ and its harmonics. The bandwidth can be defined as independent of the center frequency to be selected, e.g., constant, or as dependent on the center frequency as with bandpass filters with a quality factor. In the latter case, the frequency bandwidth Δf of each harmonic, thus, varies with the harmonic frequency according to:

(7) $$Q=\frac{f_{detect}}{\Delta f}.$$

In this way, all the bins whose frequencies are in the $\left[f_{detect}*i*\left(1-\frac{1}{2Q}\right);f_{detect}*i*\left(1+\frac{1}{2Q}\right)\right]$ band, $i=1,\ldots ,n_{h}$, are taken to calculate the energy. Table 1 gives the maximum quality factor $\frac{f_{detect}}{\Delta f_{min}}$ depending on the frequency step $\Delta_{FFT}$ (between adjacent spectral lines) given by the number of points used in the FFT.

## 5. Localization of a Moving Source: Simulation and Experiment

In this section, the case of a moving source is studied, first with a simulated source, then with the real flight of the quadcopter DJI Phantom IV. The goal is to show the relevance of the proposed method but also to see the influence of tracking on the localization performance. The effect on drone localization of the area taken into account around the harmonics in the time-frequency representation is investigated. SHC is chosen as the pitch tracking algorithm.

### 5.1. Classical DSB versus TFR Approach for Source Localization: Simulation

**Figure 11:**
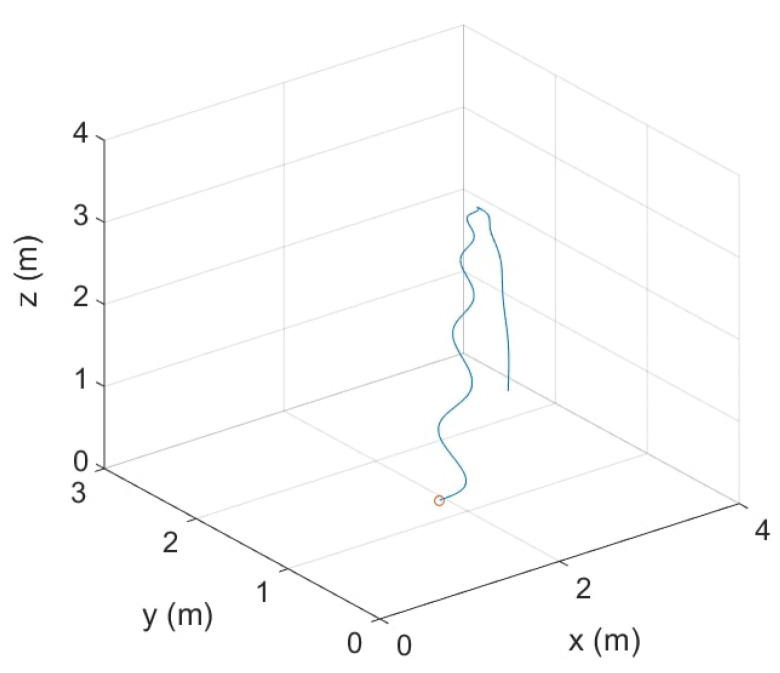
Simulated trajectory of the drone in Cartesian coordinates (the circle represents the starting position of the drone).

For this simulation, the drone starts on the ground with a distance from the antenna around 2 m, rises at a maximum height of 3.1 m, then goes down to the ground at a distance of 4 m from the antenna. Figure 11 shows the simulated trajectory in 3D Cartesian coordinates. During a real flight, the drone can adjust its rotor rotation speed so that the frequency content can vary slightly. Thus, in this simulation, a frequency modulation is implemented. The drone signal is simulated with a SNR of 5 dB. Time domain beamforming as well as SHC is provided on windows of 4096 samples from simulated signals of 5000 points to be sure to process the 4096 delays.

**Figure 12:**
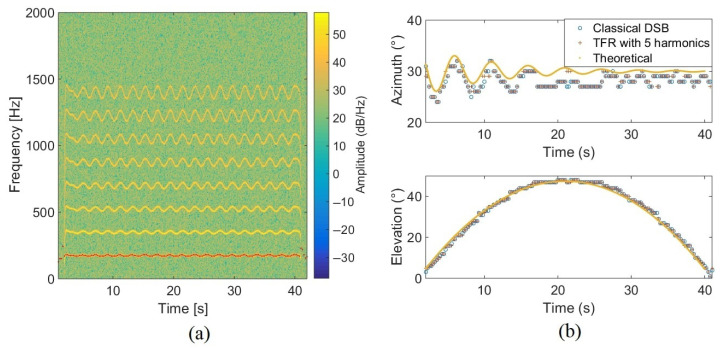
Spectrogram of the simulated source with the estimated frequencies in red (**a**). Evolution of the azimuth and elevation in time with the classical DSB, the TFR, and the theory (**b**).

Figure 12 shows the simulation results for localization. The first subfigure exhibits the estimated blade passing frequencies with SHC over time and their harmonics, the second subfigure presents the localization results provided by the proposed approach with 5 harmonics, the classical approach, and the reference position. The TFR approach is performed with a bandwidth dependent center frequency with *Q* = 10. Subfigure (a) also highlights the simulated frequency modulation: $f_{bp}$ fluctuates around 175 Hz ($f_{0}$ is around 87.5 Hz). The fundamental frequencies are well estimated and the classical and TFR-based methods give a precision error around 1.7° in azimuth and 1.1° in elevation. However, the TFR-based method performs slightly better than the classical approach, both in azimuth and elevation, according to the upper part of Table 2 dedicated to the localization performance in the simulated trajectory.

### 5.2. Influence of the Frequency Bandwidth in the TFR on Localization: Moving Drone

**Figure 13:**
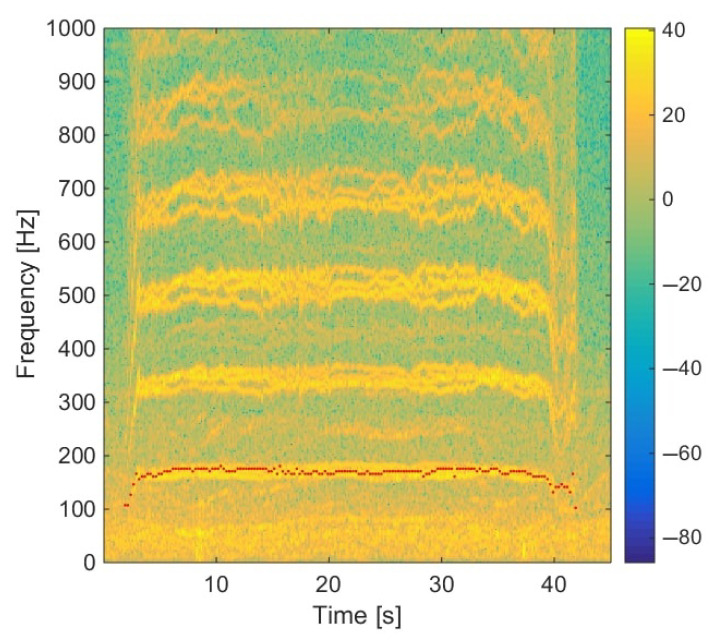
Spectrogram of the experimental trajectory (the red points are the estimated fundamental frequencies detected by SHC corresponding to $f_{bp}$).

**Figure 14:**
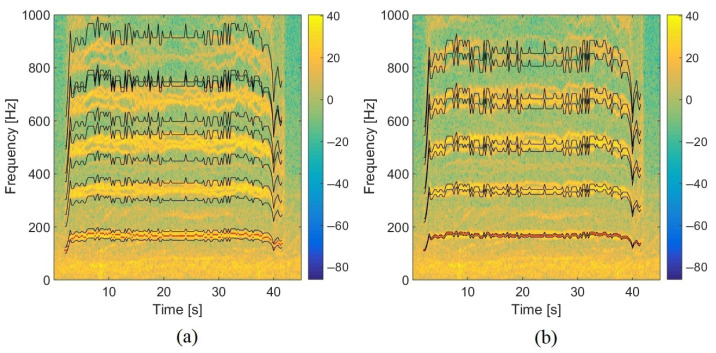
Spectrogram of the experimental trajectory with the selected content in the TFR for (**a**) *Q* = 5 and (**b**) *Q* = 17.5 (the red points are the estimated blade passing frequencies detected by SHC).

**Figure 15:**
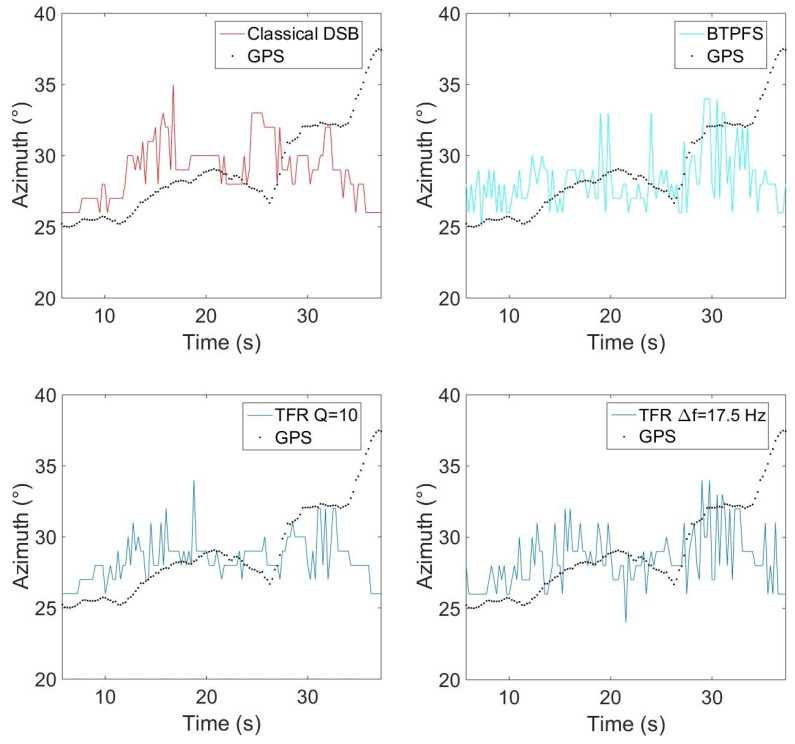
Evolution of the azimuth with time for the experimental trajectory, for the two types of bandwidth, with *Q* = 10.

**Figure 16:**
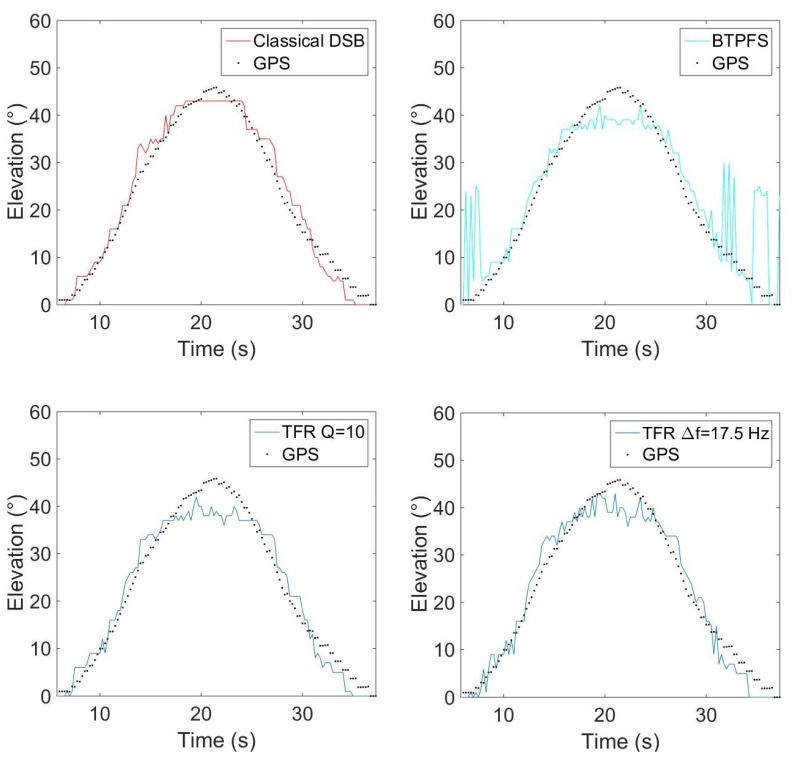
Evolution of the elevation with time for the experimental trajectory, for the two types of bandwidth, with *Q* = 10.

**Figure 17:**
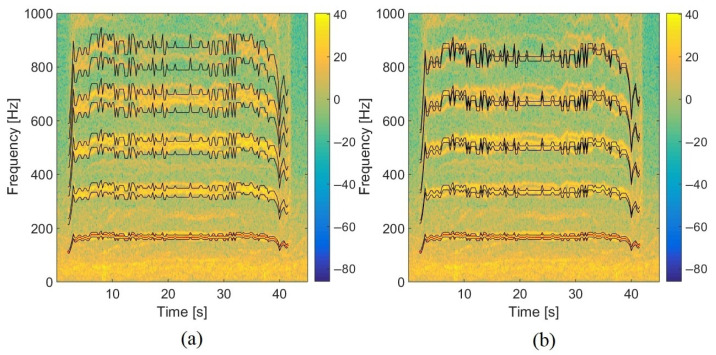
Spectrogram of the experimental trajectory highlighting the selected content in the TFR for *Q* = 10 (**a**) with the bandwidth varying with the center frequency, (**b**) with the constant bandwidth around harmonics (the red points are the estimated blade passing frequencies detected by SHC).

To evaluate the influence of the frequency bandwidth extracted in the TFR, experimental tests have been carried out with the Phantom IV drone from the company DJI on a field of Le Mans University. The array used for recording the data has the geometry given in Figure 1 and is equipped with 10 BSWA Technology MPA 416 1/4 in. microphones (20 Hz–20 kHz). The acquisition system is based on a PXI-1036 chassis from National Instruments connected to a laptop computer. The acoustic signals are recorded simultaneously on ten channels using a sampling frequency of 20 kHz. The trajectory is similar to that in the simulation presented previously. The drone starts on the ground at a distance of 3 m from the antenna, rises with a moderated speed until a height of 3.2 m, and then goes down to the ground. The azimuth and elevation angles of the drone are deduced from the embedded global positioning system (GPS). Note that the GPS has an incertitude around 3 m, which should not, however, be too penalizing for the studied trajectory. The localization performance was also examined by integrating a variable number of harmonics (3, 4, 5, 10, 15, and 20) in the power computation. With this trajectory, the errors do not evolve much (from 2.7° to 4.2° in azimuth and 12.8° to 15.6° in elevation), but the results are better when considering 4 or 5 harmonics, which is a reasonable compromise between localization accuracy and computation cost. The spectrogram of the center microphone is presented in Figure 13 with the detected frequency $f_{detect}$ in red corresponding to $f_{bp}=175$ Hz. Windows of 4096 points are used for SHC. It shows the tracks due to the four rotors running at different speeds. These tracks are compact in low frequencies but more spread out at higher frequencies. Figure 14a,b show the areas involved in the energy calculation for *Q* = 5 and *Q* = 17.5. For *Q* = 5, almost all of the useful energy appears to be extracted, while for *Q* = 17.5, the area is more selective and traces of a motor emission are found outside. Several tests were conducted with different quality factors and also constant frequency bands. They were evaluated by comparing the temporal evolution of the localization in azimuth and elevation with the reference given by the GPS. On the tested trajectory, the differences are not very marked, the results always remaining quite correct. Figure 15 and Figure 16 show the localization results for *Q* = 10 and for Δf = 17.5 Hz, which corresponds to the bandwidth around the first harmonic for *Q* = 10. A grid resolution of (1°, 1°) is used for the different localization methods and beamforming is provided on windows of 4096 points. Figure 17a,b highlight the areas considered in the time-frequency plane in these two cases. The lower part of Table 2 also contains the results of the experimental flight in terms of mean steering errors and standard deviations for the different approaches tested. The TFR method in azimuth gives deviations around 2.3° on average for both bandwidths, which is slightly lower than classical DSB (see Figure 15). The constant bandwidth allows for fewer localization errors towards the end of the trajectory. In elevation (see Figure 16), the constant bandwidth allows to have more estimates close to the reference position for elevations greater than 40°. On average, the error is slightly more important than in azimuth and classical DSB performs better, but the TFR method is still close. To have an idea of the computational cost, the algorithm speed has been measured for the TFR approach on one slot. It is computed using Matlab^®^ on a 3.20 Ghz processor (AMD Ryzen^™^ 7 5800H) and takes 10.7 s to provide a DOA estimate on a 5000-point slot with a $\left(4^{\circ },2^{\circ }\right)$ resolution. As said in the introduction, this computation time can be reduced by carrying out parallel operations. The algorithm can also be optimized by reducing the grid search around the previous estimate, assuming that it is relevant.

## 6. Conclusions and Perspectives

This paper presents a processing chain to localize a moving UAV based on its acoustic signature. It presents a good robustness to noise by the use of a microphone array associated with the technique of time domain Delay and Sum Beamforming. The process improves the method of Ref. [19], based on temporal filtering before the beamforming stage, which has the disadvantage of requiring the use of zero-phase filters, whose implementation is not inherently in real time. Here, the processing is based on the time-frequency representation of the focused signal, i.e., the beamformer’s output and could be carried out in real time. It allows selecting only the content related to the UAV’s signature to calculate the energy for each 2D position in the azimuth-elevation plane, whose maximum will give the searched drone’s DOA. It has been shown that this method gives good precision while taking only few spectral contents of the source. However, the use of several harmonics (for example, 5) improves the results in the presence of strong noise. The fact of being able to choose freely in the time-frequency plane the interesting components gives a great flexibility to the method, which is also encouraging when it is necessary to locate several sources simultaneously. The tested trajectory highlighted the interest of the pitch tracking algorithm by Spectral Harmonic Correlation and showed that taking a few frequencies around the harmonics of the blade passing frequency was effective for accurate drone localization. The method deserves to be tested on more complex trajectories with several drones in flight. Efforts could also be made to adapt the geometry of the microphone array in order to improve the localization performance in certain positions.

## Figures and Tables

**Table 1:** Maximum quality factors depending on the frequency resolution and the central frequency (sampling frequency: $f_{s}=20$ kHz, $N_{FFT}$: number of points in the FFT, $\Delta_{FFT}$: frequency step).

$N_{\mathrm{FFT}}$	Duration (s)	$\Delta_{\mathrm{FFT}}$ (Hz)	$\Delta f_{\min}$ (Hz)	$Q_{\max}$	$Q_{\max}$
				(*f* = 175 Hz)	(*f* = 350 Hz)
16,384	0.8	1.22	3	58	116
8192	0.4	2.44	5	35	70
4096	0.2	4.88	10	17.5	35
2048	0.1	9.76	20	8.75	17.5
1024	0.05	19.53	40	4.37	8.75

**Table 2:** Steering errors: mean (μ) and standard deviation (σ) for the azimuth (φ) and elevation (θ) in the simulated and experimental trajectories.

	Azimuth φ (°)		Elevation θ (°)	
	μ	σ	μ	σ
Simulated Trajectory				
Classical DSB	1.8	0.8	1.1	0.7
TFR with 5 harmonics	1.7	0.8	1.1	0.7
**Experimental Trajectory**				
Classical DSB	2.9	2.6	2	1.5
TFR with 5 harmonics (*Q* = 10)	2.3	2.5	2.8	1.9
TFR with 5 harmonics (Δ*f* = 17.5 Hz)	2.3	2.5	2.6	1.9
BTPFS	2.5	2.4	4.7	6.1
